# Appendicitis during the COVID-19 pandemic: lessons learnt from a district general hospital

**DOI:** 10.1186/s12893-021-01231-1

**Published:** 2021-05-12

**Authors:** Heather C. M. Pringle, Urszula Donigiewicz, Melissa-Rose Bennett, Eleanor Walker, George E. Fowler, Sunil Narang, Susan Ball, Robert M. Bethune

**Affiliations:** 1 Royal Devon & Exeter Hospital, Exeter, EX2 5DW Devon UK; 2 University of Exeter Medical School, St. Luke’s Campus, Heavitree Road, Exeter, EX1 2LU Devon UK; 3 NIHR Applied Research Collaboration, South West Peninsula (PenARC), University of Exeter Medical School, Exeter, EX1 2LU Devon UK

**Keywords:** Appendicitis, Coronavirus, General Surgery

## Abstract

**Background:**

The COVID-19 pandemic dramatically influenced the delivery of healthcare. In line with the UK Royal Colleges’ advice the management of acute appendicitis (AA) changed with greater consideration for non-operative management (NOM) or open appendicectomy when operative management (OM) was sought. We describe our experience of the presentation, management and outcomes for these patients to inform care for future viral pandemics.

**Methods:**

This retrospective, cohort study compared patients diagnosed with AA between March and July 2019 with those during the pandemic period of March to July 2020. Medical records were reviewed to obtain demographics, inflammatory markers, imaging, severity, management, histology, length of stay (LOS) and 90-day outcomes.

**Results:**

There were 149 and 125 patients in the 2019 and 2020 cohorts respectively. 14 patients (9.4%) had NOM in 2019 versus 31 (24.8%) in 2020 (*p* = 0.001). In the 2019 operative management (OM) group 125 patients (92.6%) had laparoscopic appendicectomy versus 65 (69.1%) in 2020. 59 patients (39.6%) had a CT in 2019 versus 70 (56%) in 2020. The median LOS was 4 days in 2019 and 3 days in 2020 (*p* = 0.03). Two patients in each year who received NOM had treatment failure (14.3% in 2019 and 6.5% in 2020). Three patients in 2019 who received OM had treatment failure (2.2%). Of 95 patients tested for COVID-19 all but one tested negative.

**Conclusion:**

During the COVID-19 pandemic there was no observed increase in severity of AA, patients had a shorter LOS and were more likely to have imaging. NOM proportionally increased with no observed change in outcomes.

**Supplementary Information:**

The online version contains supplementary material available at 10.1186/s12893-021-01231-1.

## Background

Coronavirus Disease 2019 (COVID-19), caused by the severe acute respiratory syndrome coronavirus 2 (SARS-CoV-2), was declared a pandemic by the World Health Organisation on 11th March 2020 [1]. During March restrictions were put in place on movement and social gatherings in an attempt to control viral spread. This culminated in United Kingdom (UK) wide ‘lock-down’ on the 24th March which led to a reduction in all emergency admissions to hospitals across England [2]. A report from Italy noted a reduction in presentation of acute appendicitis (AA) and worsening severity on presentation [3]; so far undocumented in the UK.

On 26th March 2020 the UK Joint Royal Colleges published guidance [4] for surgeons working during the COVID-19 pandemic advising considerable caution for all laparoscopic surgery due to the potential risk associated with aerosol formation on deflation of the pneumoperitoneum as well as the smoke plume produced with laparoscopic electrocautery and with high-energy devices. The Colleges also advised non-operative management (NOM) to be used in selected cases and if operative management was necessary, an open appendicectomy should be the method of choice.

Acute appendicitis is the most common surgical emergency. The classic clinical presentation is present in approximately one third of patients with many in the extremes of age presenting atypically. Misdiagnosis in these age groups is not rare and can lead to an increased rate of complications [5]*.* To avoid complications and the associated morbidity early diagnosis and treatment of AA is essential.

Prior to the pandemic the general feeling in our Trust was that laparoscopic appendicectomy was the preferred surgical option over an open approach for the management of AA. Part of the reason for this could be that open appendicectomy has been associated with greater post-operative pain, longer hospital admissions and a slower return to normal physical activity [6]. Further to this, in our Trust NOM was previously felt to be reserved for cases where it was the patient’s choice or OM was unfeasible in the clinical situation. There is considerable controversy in the literature regarding the role of NOM as the first line treatment for AA [7]. NOM for AA at five years compared to a primary appendicectomy shows a significantly lower post-intervention complication rate by avoiding surgical site infection, incisional hernias, post-operative abscesses, post-operative ileus and other complications [8]. While conflicting evidence shows there is a non-significantly higher rate of complicated appendicitis with delayed surgery in patients receiving NOM as first-line therapy [8] and of the patients who have NOM for AA there is a 39% risk of recurrence and 27.3% will eventually require an interval appendicectomy [9, 10]. This highlights some of the controversy that can be found in the literature.

During the early stages of the COVID-19 pandemic, surgical practice changed for patients with AA as units gave greater consideration to the option of NOM and open appendicectomy where OM was sought. We describe the changes to the management of AA in our Trust in response to advice by the Royal Colleges, to explore the impact these changes had on short-term outcomes and to provide insight on some of the lessons we have learned as we face the second wave.

## Methods

We undertook a single-centre retrospective cohort analysis to compare the management of AA both before and during the COVID-19 pandemic. The Royal Devon and Exeter Hospital is a University-affiliated NHS Foundation Trust with 800 beds offering secondary care specialist services to a population of 460,000 people.

All patients discharged with a diagnosis of acute appendicitis during the two study periods were included. Any patients relocating to a tertiary centre during their treatment journey and any patients in which acute appendicitis was not the primary diagnosis were excluded.

Data were obtained for all patients from the 1st of March to the 31st of July 2019 and the equivalent five-month period in 2020. The 2019 cohort served as a control for comparison. Electronic health records (EHR) were interrogated to obtain baseline demographics, patient comorbidities as represented by the American Society of Anaesthesiologists (ASA) physical status classification [11], admission blood tests (C-reactive protein (CRP), White Cell Count ((WCC) and platelets), imaging investigations (ultrasound scan (USS) or computed tomography (CT) with or without contrast enhancement) and outcome data. Management strategy was defined as one of the following: non-operative management (NOM) with antibiotics; operative management (OM) (with laparoscopic appendicectomy or open appendicectomy).

Operation notes were analysed to grade the severity of intra-operative findings in AA according to a nationally recognised system shown in Table 1 [12]. Other information extracted from the EHR included: hospital length of stay (LOS); post-operative complications; re-attendance to hospital within 90 days from the initial presentation. Symptoms were reviewed daily by medical staff and patients were classified as being symptomatic or asymptomatic. COVID-19 tests (for SARS-CoV-2 RNA) were taken by trained Health Care Assistants and Nurses; the tests had a sensitivity of 71–98% [13].

**Table 1:** Intraoperative appearances of acute appendicitis [12]

Non-complicated Acute Appendicitis
Grade 0—Normal looking appendix (Endoappendicitis/Periappendicitis)
Grade 1—Inflamed appendix (Hyperemia, oedema ± fibrin without or little pericolic fluid)
Complicated Acute Appendicitis
Grade 2—Necrosis Grade 2A—Segmental necrosis (without or little pericolic fluid) Grade 2B—Base necrosis (without or little pericolic fluid)
Grade 3—Inflammatory Tumour Grade 3A—Flegmom Grade 3B—Abscess < 5 cm without peritoneal free air Grade 3C—Abscess > 5 cm without peritoneal free air
Grade 4—Perforated—diffuse peritonitis with or without peritoneal free air

Histology was reported on all operatively managed cases of AA and classified as ‘normal appendix, ‘acute inflammation’, ‘tumour’ or ‘other findings’.

The primary outcome for this study was treatment failure defined as a different treatment strategy to that which was originally intended. The secondary outcomes were length of stay, re-attendance without treatment failure and post-operative complications without treatment failure.

Data on demographics, blood results and imaging modalities used are summarised for the 2019 and 2020 cohorts (there is access to the raw data in Additional file 1). The management of AA (NOM or OM) was compared between cohorts using a Chi-squared test, and blood results were compared using two sample t-tests and Mann Whitney tests. Within OM patients, surgical approaches and histology outcomes are summarised and compared between cohorts. Re-attendances are summarised and their management approaches were compared between cohorts. Length of stay was compared between cohorts using a Mann Whitney test.

Ethical approval was not required due to the retrospective and anonymised nature of the study. The project was registered locally and approved by the clinical audit department (reference number 20-4591).

## Results

A total of 274 patients were included in this study (149 in 2019; 125 in 2020). Baseline characteristics, admission blood results and imaging modalities are reported in Table 2. A smaller proportion of patients were treated with NOM in the 2019 cohort than in the 2020 cohort (14/149 (9.4%) v (29/125 (23.2%)). Two patients in the 2020 cohort were transferred to a tertiary centre specialising in paediatric surgery for their ongoing care and were excluded from the analysis.

**Table 2:** Baseline demographics, blood results and imaging modality used

	2019	2020
Number of patients	149	125
Age, in years, mean (SD)	37.2 (21.2)	38.1 (21.0)
Sex, n (%)		
Male	80 (53.7)	67 (53.6)
Female	69 (46.3)	58 (46.4)
ASA score, n (%)		
I	109 (73.2)	76 (60.8)
II	27 (18.1)	44 (35.2)
III	12 (8.1)	5 (4.0)
IV	1 (0.7)	0 (0)
Admission blood test results		
CRP (mg/L) median (range)^≠^	44 (0–494)	37 (0–447)
WCC (1 × 10^9^/L) mean (SD) ^¥^	13.3 (4.2)	12.3 (3.5)
Platelets (1 × 10^9^/L) mean (SD)^×^	253.3 (68.1)	252.4 (68.5)
Imaging modality, n (%)		
None	58 (38.9)	24 (19.2)
Ultrasound scan (USS)	32 (21.5)	31 (24.8)
Computed tomography (CT)^¥^	57 (38.3)	64 (51.2)
USS and CT	2 (1.3)	6 (4.8)
Imaging findings, n (%)		
None	58 (38.9)	24 (19.2)
Normal/inconclusive	16 (10.7)	8 (6.4)
Uncomplicated AA	58 (38.9)	70 (56.0)
Complicated AA	16 (10.7)	23 (18.4)
Other	1 (1.0)*	0 (0.0)
COVID-19	N/A	
Not tested, n (%)	149 (100)	30 (24.0)
Tested, n (%)	0 (0)	95 (76.0)
Positive		1 (1.1)
Negative		94 (98.9)

^≠^(*p* = 0.75)

^¥^(*p* = 0.03)

^×^(*p* = 0.92)

*Ovarian cyst

### Operative management (OM)

Table 3 shows surgical technique, intraoperative findings and the histology results for the OM patients. There was a statistically significant difference between the cohorts in terms of the proportion receiving NOM or OM (*p* = 0.001) and having laparoscopic or open surgery (*p* < 0.001).

**Table 3:** Acute appendicitis management and outcomes among patients treated with operative management

Operative management	2019 (N = 135)	2020 (N = 94)
Laparoscopic Surgery, n (%)	125 (92.6)	65 (69.1)
Open	10 (7.4)^±^	28 (29.7)^≠^
Unknown	0 (0.0)	1 (2.1)*
Intraoperative findings, n (%)		
Grade 0	5 (3.7)	3 (3.2)
Grade 1	72 (53.3)	55 (58.5)
Grade 2a	9 (6.7)	7 (7.4)
Grade 2b	2 (1.5)	3 (3.2)
Grade 3a	10 (7.4)	5 (5.3)
Grade 3b	18 (13.3)	7 (7.4)
Grade 3c	2 (1.5)	1 (1.1)
Grade 4	17 (12.6)	10 (10.7)
Histology, n (%)		
Acute inflammation	122 (90.4)	88 (93.4)
Tumour	1 (0.7) adenocarcinoma 3 (2.2) mucinous neoplasm 1 (0.7) neuroendocrine	0
Other	5 (3.7) normal appendix 1 (2.1) chronic inflammation	3 (3.2) normal appendix

^±^Two began as laparoscopic and were converted to open

^≠^Four began as laparoscopic and were converted to open

*Operation note unrecoverable in electronic medical records

### Morbidity and outcomes

The median length of stay (LOS) in 2019 was 4 days (interquartile range (IQR) 3 to 6 days), versus 3 days (IQR 2 to 5 days) in 2020 (*p* = *0.03)*. The patients who had NOM had a median LOS of 5.5 days (IQR 5 to 7 days) in the 2019 cohort compared to 3 days (IQR 2 to 6 days) in the 2020 cohort. The OM group had a median LOS of 4 days (IQR 3 to 5 days) in the 2019 cohort and 3 days (IQR 2 to 4.8 days) in the 2020 cohort.

In the 2019 cohort, there were two re-attendances (14.3%) in the first 90 days in the NOM group—one for an appendiceal abscess requiring interventional radiological (IR) drainage and the other for further intravenous antibiotics. Out of 135 patients in the OM group there were three reattendances (2.2%)—two patients had a post-operative collection and one had a superficial wound dehiscence.

In the 2020 cohort, two patients (6.5%) in the NOM group reattended and required a different management strategy—one had an open appendicectomy and the other received intravenous antibiotics for an appendiceal abscess confirmed on CT. In the 2020 OM group there were no reattendances requiring intervention.

COVID-19 swab tests became mandatory for all inpatients on 10th April 2020, this equated to 95 patients (76%) in the 2020 cohort who were tested. One patient tested positive (1.1%) and the remaining 94 (98.9%) who were tested were negative. The swab-positive patient was originally treated with NOM as the symptoms of acute appendicitis coincided with mild COVID-19 symptoms. The patient then reattended 14 days later and had a laparoscopic appendicectomy which showed acute inflammation on histology.

## Discussion

The COVID-19 pandemic brought unprecedented challenges to the National Health Service and drove widespread changes in healthcare delivery. The UK had the benefit of witnessing the experiences in other countries before a surge in UK COVID-19 cases, allowing our surgical services some time to prepare [3, 14]. The UK Intercollegiate guidance [4] encouraged a move towards NOM of AA where appropriate. Two recent trials support this approach despite the optimum treatment of AA remaining controversial [5, 8, 15, 16].

During the pandemic other General Surgery departments across the UK were having similar quandaries as us in decision-making processes as a result of the Royal Colleges’ advice. The HAREM study is an observational study which looked at 500 patients presenting with appendicitis during the first wave of the pandemic [15]. Their results were similar to ours showing good outcomes in all patients with AA following the intercollegiate advice and that NOM is a safe alternative to surgery in uncomplicated AA. Their study provides 30 day follow up whereas ours provides 90 day follow up. Our study adds to the literature by providing an insight into the workings behind a single centre and comparing our findings during the pandemic to previous data. This will help other similar sized centres in their decision-making for future viral pandemics.

Following nationwide ‘lock-down’ in March 2020 fewer patients attended with AA (149 in 2019 vs 125 in 2020). This finding was not significant and could be a normal variation. A larger retrospective study could consider evaluating changes in the epidemiology of AA in the COVID-19 pandemic. NOM was used more frequently (9.4% in 2019 vs 24.8% in 2020) although the majority of patients still received OM. Patient selection for NOM appears to be in keeping with the advice provided by a recent AA update which advises NOM in patients with uncomplicated AA confirmed on imaging [5]. The observed reduction in laparoscopic appendicectomy (92.6% of OM in 2019 vs 69.1% of OM in 2020; *p* < 0.001) reflects our Trust’s iteration of the Intercollegiate advice [4]. More patients re-attended following NOM in both years (14.3% of NOM and 2.2% of OM in 2019 vs 6.5% of NOM and 0% of OM in 2020). There was no adverse outcome for these patients who reattended. They had imaging, an extension of their antibiotic regime, reassurance or symptom control. This serves to make the point that although we have observed no significant morbidity with NOM there has still been use of healthcare resources and this is important to consider in the balance between NOM and OM in uncomplicated AA.

The current pandemic presents unquantified risks to patients. Some early collaborative trials showed there was an increased risk of morbidity and mortality in COVID-19 positive patients undergoing surgery [17]. For surgeons this translates to new challenges in consent and shared decision-making. As the COVID-19 outbreak progressed our consent process changed to counsel patients on the risks associated with contracting the virus during the perioperative period. In 2019, 59 patients (39.5%) had a CT compared to 70 patients (56%) in 2020. This was to improve diagnostics and to guide appropriate management reducing unnecessary operating. Clinical decision-making and available resources are part of the reason that not all cases had imaging. Recent published guidance pushes towards imaging rather than diagnostic laparoscopy [18] and we had a lower negative appendicectomy rate in both cohorts (3.7% in 2019 and 3.2% in 2020) when compared with the literature [19, 20]. There is evidence that COVID-19 infection can clinically mimic AA in atypical cases [21] but none of the patients with a negative appendicectomy had COVID-19 confirmed on swab.

Patients presenting with AA during the COVID-19 pandemic had shorter hospital stays. This was the case for patients following an operation (median of 4 days in 2019 vs 3 days in 2020) despite evidence showing that open appendicectomies result in longer hospital admissions [6]. During the pandemic there were non-clinical factors which drove prompt discharges compared to normal circumstances such as pressure from senior management to increase bed capacity as well as patient choice to reduce their risk of exposure to the virus. These findings suggest that under normal circumstances we may be able to safely discharge patients earlier who are recovering from AA. This is supported by recent evidence demonstrating that OM of AA can be conducted as a day case in 25% of patients [22].

In this study, there are several limitations. It is a retrospective study and captures a small cohort of patients from a single-centre. However, it represents data from a large district general hospital and other studies have reported similar findings [15, 16] We cannot comment if patients re-attended out of area though this seems unlikely with Government advice to avoid non-essential travel [23]. The authors would have included a comparison on the rate of delayed presentation and rate of perforated appendicitis between the two study periods. However, given the retrospective and observational nature of the study we relied on thorough clinical documentation of time of symptom onset which unfortunately was not available consistently or accurately. This is something that could be considered in similar prospective studies in the future.

In one sense the pandemic has presented an opportunity to put alternative approaches to the test. Easing of the preventive measures has demonstrated an evolving second peak of the virus and pressure on the health services may be worse than the first [24]. This study has demonstrated few complications following NOM and subsequently our Trust is open to NOM in uncomplicated AA. Along with the strengthening body of evidence in support of this management strategy we are more equipped to counsel patients to make informed decisions about their care.

## Conclusions

During the early months of the COVID-19 pandemic, we saw no increase in the severity of acute appendicitis presentations. In our department, more patients were managed without surgery, and spent less time in hospital. Consistent with other studies, we saw no significant difference in outcomes. We recognise the need for further studies to assess longer-term outcomes, but this study adds to the case for a non-operative first line approach to acute appendicitis.

## Supplementary Information

**Additional file 1.** Acute Appendicitis During COVID Dataset. Anonymised raw data including all variables collected. Patients in the dataset are only those which adhere to the inclusion criteria.

## Data Availability

All data generated or analysed during this study are included in this published article [and its additional information files which can be found after the references]. The project was registered locally and approved by the clinical audit department at the Royal Devon and Exeter Hospital NHS Trust (reference number 20-4591). Public access to the database is closed.

## Abbreviations

AA: Acute appendicitis
NOM: Non-operative management
OM: Operative management
LOS: Length of Stay
COVID-19: Coronavirus Disease 2019
SARS-CoV-2: Severe acute respiratory syndrome coronavirus 2
UK: United Kingdom
HER: Electronic health records
ASA: American Society of Anaesthesiologists
CRP: C-reactive protein
WCC: White Cell Count
USS: Ultrasound Scan
CT: Computed Tomography
IQR: Interquartile range
IR: Interventional radiological
